# Connectivity Restoration in Wireless Sensor Networks via Space Network Coding

**DOI:** 10.3390/s17040902

**Published:** 2017-04-20

**Authors:** Alfred Uwitonze, Jiaqing Huang, Yuanqing Ye, Wenqing Cheng

**Affiliations:** 1 School of Electronic Information & Communications, Huazhong University of Science & Technology, 1037 Luoyu Road, Hongshan District, Wuhan 430074, China; alfred@mail.hust.edu.cn (A.U.); chengwq@mail.hust.edu.cn (W.C.); 2 Department of Electrical & Computer Engineering, Carnegie Mellon University, 5000 Forbes Ave, Pittsburgh, PA 15213, USA; yuanqiny@andrew.cmu.edu

**Keywords:** connectivity restoration, wireless sensor networks, network partitioning, space network coding, Delaunay triangulation, relay node placement

## Abstract

The problem of finding the number and optimal positions of relay nodes for restoring the network connectivity in partitioned Wireless Sensor Networks (WSNs) is Non-deterministic Polynomial-time hard (NP-hard) and thus heuristic methods are preferred to solve it. This paper proposes a novel polynomial time heuristic algorithm, namely, Relay Placement using Space Network Coding (RPSNC), to solve this problem, where Space Network Coding, also called Space Information Flow (SIF), is a new research paradigm that studies network coding in Euclidean space, in which extra relay nodes can be introduced to reduce the cost of communication. Unlike contemporary schemes that are often based on Minimum Spanning Tree (MST), Euclidean Steiner Minimal Tree (ESMT) or a combination of MST with ESMT, RPSNC is a new min-cost multicast space network coding approach that combines Delaunay triangulation and non-uniform partitioning techniques for generating a number of candidate relay nodes, and then linear programming is applied for choosing the optimal relay nodes and computing their connection links with terminals. Subsequently, an equilibrium method is used to refine the locations of the optimal relay nodes, by moving them to balanced positions. RPSNC can adapt to any density distribution of relay nodes and terminals, as well as any density distribution of terminals. The performance and complexity of RPSNC are analyzed and its performance is validated through simulation experiments.

## 1. Introduction

Recent years have witnessed a massive growth in the use of Wireless Sensor Networks (WSNs) in numerous applications, especially those operating in hostile environments such as space exploration, disaster management, search and rescue, and battlefield surveillance [1]. In some of these applications that operate in such a hostile setup, a set of sensor nodes are deployed to an area of interest to collaboratively monitor certain events of interest. By deploying the sensor nodes to operate in harsh environments, it would be possible to avoid risk to human life and reduce the application cost [2]. Sensors are small and battery operated devices, having limited processing and communication capabilities  [3].

Upon deployment, the sensor nodes are expected to stay reachable to each other and form a network. Network connectivity enables sensor nodes to share data and to coordinate their actions while performing a task, and to forward the collected data to a command center [4]. Therefore, the inter-sensor nodes connectivity should be maintained all the time to enable such interactions.

However, the nodes of WSNs that serve in harsh environments are susceptible to failure. For example, in a battlefield, parts of the deployment area may be bombed, destroying the sensor nodes in the vicinity. The failed nodes may cause the WSN to be split into multiple disjoint partitions due to the loss of connectivity among the partitions. Losing the network connectivity in WSNs has a very negative effect on the applications since it prevents data exchange and hinders the coordination among some sensor nodes. Given the importance of data sharing in achieving WSN application goals, restoring the connectivity among these WSN partitions would be very necessary so that the WSN becomes operational again. The Euclidean Steiner Minimal Tree (ESMT) [5] based approach for deploying relay nodes is one of the most efficient approaches [6] for restoring the connectivity in such a case. Relay nodes are usually assumed to be more powerful and expensive than sensor nodes. Given hardware and deployment cost associated with relay nodes, it is usually desirable to minimize the number of required relay nodes. Thus, the goal of the recovery process in this paper is to re-establish the connectivity by forming an inter-partitions topology, while minimizing the number of required relay nodes. Such relay nodes placement optimization problem is proven to be an NP-hard problem by Lin and Xue [7].

Several heuristic approaches have been proposed in the literature to solve this problem. Some of these heuristics focused on minimizing the count of the required relay nodes by forming a Minimum Spanning Tree (MST), in which every relay node has a node degree of two [7,8,9]. Meanwhile, other works focused on the degree of connectivity of the formed topology with less priority on the number of relay nodes required [4,10,11]. In this paper, we propose a novel relay nodes placement strategy based on space network coding that minimizes the number of required relay nodes to restore the connectivity of a partitioned WSN. In the context of this paper, each partition is represented by one sensor node that we call terminal.

**Figure 1:**
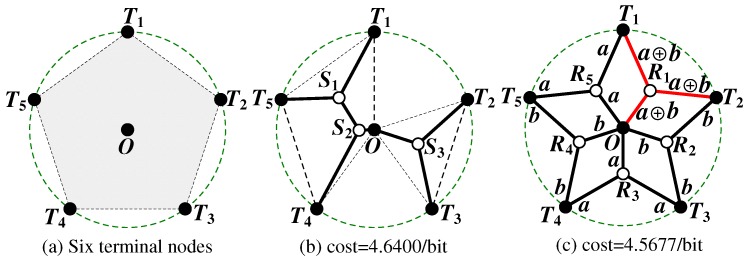
A Pentagram example. (**a**) Six terminal nodes in 2D Euclidean space; (**b**) optimal solution by ESMT; (**c**) optimal solution by space network coding.

Departing from *Network Information Flow* (NIF) [12] proposed in 2000, in which extra relay nodes can not be introduced, *Space Information Flow* (SIF) [13], also called Space Network Coding, is a concept proposed in 2011 that studies network coding in Euclidean space, in which an extra set of relay nodes can be inserted to connect a given set of terminal nodes. This paper uses the terms SIF and space network coding in an interchangeable way. The goal of SIF is to minimize the *cost* of constructing a network, where the *cost* is defined as the total length of information transmission required to achieve a one-bit end-to-end multicast throughput [14]. A *Pentagram* [15] example illustrated in Figure 1 demonstrates that the performance of SIF can be strictly better than that of ESMT, with the *Cost Advantage* (CA) [13] being strictly bigger than 1. CA is defined as the ratio of minimum cost necessary for achieving a target throughput by routing over that of network coding. Figure 1a depicts a multicast version of SIF problem with six terminal nodes in a 2D Euclidean space, where five nodes ($T_{1}$ to $T_{5}$) are evenly distributed along a circle and form a regular pentagon centered at node *O*. The radius of the circumscribed circle is 1. Node *O* is chosen as the multicast source, with the other five being receivers. We compare network coding and routing, using cost as our comparison metric. Routing in space with additional relay nodes allowed to be introduced, in order to connect the terminal nodes, is equivalent to the ESMT problem. With ESMT, the optimal solution is computed by ESMT exact algorithm [16], which has a cost of 4.6400/bit, as illustrated in Figure 1b. Three Steiner nodes ($S_{1}$ to $S_{3}$) are inserted to connect the terminal nodes, each adjacent to three links that form three angles of $120^{\circ }$. Figure 1c shows the optimal solution based on space network coding, where five relay nodes ($R_{1}$ to $R_{5}$) are inserted to connect the terminal nodes, each adjacent to three links that form three angles of $120^{\circ }$. The total distance is 9.1354 and each sink receives two bits. The normalized per bit cost is 9.1354/2 = 4.5677/bit, and it is strictly smaller than the optimal cost based on ESMT. CA = 4.6400/4.5677 ≈ 1.0158 > 1. Despite its small value, we emphasize that the gap between the two optimal costs reveals that space network coding is fundamentally a different problem from routing in space, with a different problem structure, and probably a different computational complexity.

The main contributions of this work are as follows. Unlike most existing works that are based on MST or ESMT, we propose a new polynomial time min-cost multicast Relay Placement algorithm based on Space Network Coding (RPSNC) that optimally solves the problem of restoring the network connectivity for the special case of three terminals and strives to minimize the number of relay nodes required for restoring the connectivity for larger networks using Delaunay triangulation, non-uniform partitioning and linear programming techniques. Delaunay triangulation is able to adapt RPSNC to any density distribution of relay nodes and terminals, non-uniform partitioning aims to adapt RPSNC to any density distribution of terminals, while linear programming aims to optimize the required number of relay nodes by computing the min-cost of the inter-partitions topology. Linear programming also determines the connection links between the relay nodes and terminals. It is worth noting that the proposed RPSNC computes not only the minimal number of relay nodes, but also the geometric location of each required relay node in Euclidean space. To the best of our knowledge, this is the first work to propose a min-cost multicast space network coding based algorithm for connectivity restoration in WSNs.

RPSNC first selects one representative sensor node in each partition and sets it as a terminal. It then combines non-uniform partitioning and Delaunay triangulation techniques for generating the candidate relay nodes; afterwards, RPSNC applies linear programming for choosing the optimal relay nodes and computing their connection links with terminals. Basically, the distance between the optimal relay nodes and terminals may exceed the communication range of the optimal relay nodes. Hence, RPSNC opts to populate additional relay nodes along the connection links to achieve a strong connectivity and a better relay nodes coverage. The simulation results demonstrate the effectiveness of RPSNC.

The rest of this paper is organized as follows. Related work is covered in Section 2. The problem definition and considered system model are described in Section 3. The details of RPSNC are provided in Section 4. The simulation results are presented in Section 5. The paper is finally concluded in Section 6.

## 2. Related Work

Relay nodes placement in WSNs has been used not only for restoring the connectivity, but also for improving the WSNs performance, such as WSN longevity [17]. Given the scope of contributions of this paper, this section focuses on works which target the connectivity restoration in partitioned WSNs. Some of the published works in this category employ centralized algorithms [18,19], where one of the sensor nodes is in charge of generating the recovery plan and coordinating the relocation process. These approaches rely on the availability of an alternate communication path to inform other nodes on what to do. On the other hand, distributed algorithms [20,21] have been the preferred choice for restoring the connectivity of large networks and for repairing partitioned networks. In such a case, the sensor nodes are assumed to have some pre-failure state (e.g., k-hop) information and utilize that information to detect and recover from network partitioning. However, these algorithms may not be well suited for restoring the connectivity when the network is significantly damaged due to the fact that sensor nodes have resource and capability constraints and may not be able for a long distance move if the affected area is large. Most of the published studies on connectivity restoration in partitioned WSNs strive to restore the connectivity by carefully placing a minimum number of relay nodes in such a way that data routes are formed between each pair of terminals. As mentioned earlier, this problem is proven to be NP-Hard by Lin and Xue [7]. They proposed a polynomial time approximation algorithm which populates relay nodes on the edges of MST of the terminals. First, the algorithm constructs a complete graph G=(V,E) of terminals, where *V* is the set of terminals and *E* is the set of all edges (u,v), where u,v∈V. Subsequently, using Kruskal’s MST algorithm, the tree edges are computed. Relay nodes are then deployed along each edge in the tree at a distance of at most *R* apart, where *R* is the communication range of a relay node.

Wang et al. [22] proposed a Collaborative Connectivity Restoration Algorithm (CCRA) based on cooperative communication and node maneuverability to restore the network connectivity after multiple nodes failure. CCRA opts to simplify the recovery process by gridding. Moreover, the distance that an individual node needs to travel during the recovery process is reduced by choosing the nearest suitable candidates. Cheng et al. [8] proposed a three-step heuristic algorithm to form ESMT with minimum Steiner points. In the first step, it connects the sensor nodes where the distance between the sensor nodes is less than or equal to *R*. In the second step, it forms three-stars in that, for each subset of three sensor nodes {u,v,w}, there exists a point *s* such that *s* is at most *R* units away from *u*, *v* and *w*. In the last step, the algorithm populates relay nodes along the MST edges connecting two different connected components.

The literature works [3,6,9] have studied the same problem that we tackle in this paper. In [3], Senel and Younis presented a three-step algorithm called Federating network Segments via Triangular Steiner tree Approximation (FeSTA), which is based on the triangle Steiner method. In the first step, FeSTA finds the best triangles and forms islands of segments by establishing intra-triangle connectivity. Then, in the second step, disjoint islands of segment are federated. In the final step, the Steiner nodes of the best triangles are applied for optimization. In [6], Chen and Shi proposed a step by step heuristic algorithm called Quadrilateral Steiner Tree Algorithm (QTA), which is based on the quadrilateral Steiner method. First, the disjointed partitions are detected and their locations are determined. Then, the appropriate quadrilaterals are selected to connect the partitions and the Steiner nodes of these quadrilaterals are found. The partitions that are not connected by the Steiner nodes of the selected quadrilaterals are connected with the MST method. Unlike FeSTA and QTA, which are based on triangle and quadrilateral Steiner methods, respectively, RPSNC is based on space network coding and takes full advantage of considering Delaunay triangles and quadrilaterals Steiner nodes. In contrast to FeSTA and QTA that connect the partitions (terminals) of WSN step by step, RPSNC connects all the partitions at once, which may enable us to reduce the number of required relay nodes. Furthermore, RPSNC significantly performs better than FeSTA and QTA, as it will be proven and confirmed later by RPSNC analysis and the simulation experiments. In [9], Chen et al. proposed an algorithm called a Minimum Spanning Tree based on a single-Tiered Relay Node Placement (MST_1TRNP), which deploys relay nodes along MST edges of the terminals, where terminals can be considered as sensor nodes. Unlike MST_1TRNP, RPSNC finds the Delaunay triangles and concatenates two neighboring Delaunay triangles to obtain quadrilaterals. Then, RPSNC places the relay nodes at the Steiner points inside the Delaunay triangles and quadrilaterals.

The focus of [4,10,11] is also on a variant of the problem of re-establishing the connectivity of a partitioned WSN, where additional metrics of relay node degree must be optimized. In [4], Lee and Younis opt to federate the disjoint partitions in a such way that the inter-partition topology has a high node degree. They modeled the deployment area as a grid of equal-sized cells, and each network partition is assumed to be located in the middle of the cell. A Cell-based Optimized Relay node Placement (CORP) algorithm is proposed. CORP is a two-phase polynomial time algorithm. The first phase aims to iteratively identify the border terminals and the best cell to deploy a relay node. The second phase aims to connect all terminals by populating relay nodes inwards until all relay nodes become reachable to one another, after which redundant relay nodes are pruned. In [10], Zheng et al. proposed a Partial 3-Connectivity Restoration Algorithm (P3CRA) that constructs the dual-ring topology structure to improve the quality of the formed topology. P3CRA ensures that, after the connectivity restoration, all the partitions have at least three-connectivity, and the deployed relay nodes have at least two-connectivity. In [11], Senel et al. proposed a SpiderWeb approach that opts to re-establish the connectivity using the least number of relay nodes, while achieving a high node degree in the formed topology. As opposed to these approaches, minimizing the number of required relay nodes is the main goal for RPSNC. A comparative summary of some approaches for relay nodes placement is provided in Table 1.

Ahmadi et al. [24] focused on the problem of simultaneously reducing energy consumption and increasing WSN lifetime. They proposed an effective algorithm that provides greater efficiency energy consumption as it preserves the network with its low energy consumption. In a subsequent work, Naranjo et al. [25] proposed a modified Stable Election Protocol (SEP), named Prolong-SEP (P-SEP) to prolong the stable period of Fog-supported WSNs by maintaining the balanced energy consumption. Unlike these works that are based on routing, we propose a min-cost multicast relay placement approach based on space network coding for restoring the connectivity in partitioned WSNs.

In line with space network coding, Xiahou et al. [26] applied space network coding as a tool to design a framework for analyzing the network coding conjecture in undirected graphs.

Regarding the applications of network coding in WSNs, Wang et al. [27] applied partial network coding for data collection in WSNs operating in harsh environments. Rout and Ghosh [28] proposed a network coding based communication algorithm to improve the WSN lifetime. Eritmen and Keskinoz [29] proposed a signature selection and relay power allocation method based on network coding that significantly improves the throughput of WSNs that operate over non-orthogonal channels. Ayday et al. [30] proposed a network coding based protocol called Location-Aware Network-Coding Security (LANCS) that provides security services such as data confidentiality, authenticity, and availability in WSNs.

However, to the best of our knowledge, space network coding has not been applied for connectivity restoration in WSNs. Our work is different from the above works in a significant aspect: we introduce a new min-cost multicast space network coding approach that aims to solve the problem of restoring the network connectivity in partitioned WSNs.

## 3. Problem Statement and Fundamental Definitions

In the context of this paper, a WSN is a set of sensors scattered in an area of interest to detect and track some events. We consider a WSN that has been split into multiple disjoint partitions due to a major scale damage in a part of the network, e.g., inflicted by explosives in a battlefield. We strive to restore the partitioned network by linking the disconnected partitions. A sensor is battery-operated and has limited communication and processing capabilities. We assume that sensors are stationary, which is typical for WSNs. All communication is over a single shared wireless channel. A wireless link can be established between a pair of sensor nodes if they are within the communication range *R* of each other.

A relay node is a more powerful node with significantly more energy reserve, longer transmission range and richer processing capabilities than sensors. Although relay nodes can be equipped with sensing circuitry, they mainly perform data aggregation and forwarding. Unlike sensors, relay nodes can be mobile and have some navigation capabilities. Relay nodes are favored in the recovery process since it is easier to accurately place them relative to sensors and their communication range is larger, which facilitate and expedite the connectivity restoration among the disjoint partitions. Intuitively, relay nodes are more expensive and consequently the main objective of this paper is to minimize the required number of relay nodes. In this paper, it is assumed that all deployed relay nodes have the same communication range “*R*”.

The problem that we study in this paper can be formally defined as follows: “Given N≥3 disjoint partitions of a WSN, find the minimum number and optimal positions of required relay nodes for restoring inter-partitions connectivity”. This problem is challenging in that we need to compute not only the number of optimal relay nodes and the geometric location of each optimal relay node, but also the best way of connecting them with terminals to achieve the optimal solutions. As mentioned earlier, we assume that each partition is represented by a sensor node, which we call terminal. Each terminal connects a partition $P_{i}$ to the resulting topology and plays a role of gateway after restoration. In the context of this paper, a partition is a connected set of sensor nodes, denoted as $P_{i}$.

**Definition** **1.** Let N denotes the planar points. The Voronoi diagram of N partitions the plane into regions, called Voronoi regions, such that each point $p_{j}\in N$ lies in exactly one region. The Voronoi polygon of a point $p_{j}$, denoted as $VP(p_{j})$, consists of all points in the plane for which $p_{j}$ is the closest point among all other points. The collection of Voronoi polygons $VP(p_{j})$ for each $p_{j}\in N$ is called Voronoi diagram VD(N). The Delaunay triangulation DT(N) is the planar straight line dual graph of Voronoi diagram VD(N) [31].

**Definition** **2.** *The number of relay nodes required for connecting two terminals u and v is called the e-weight of the edge $\bar{uv}$ and denoted as*

$$\begin{array}{c} W_{e}\left(u,v\right)=\left\lceil \frac{\left|uv\right|}{R}\right\rceil -1, \end{array}$$

*where |uv| is the Euclidean distance between u and v, and R is the communication range of relay nodes.*

**Definition** **3.** *Let $T_{i}\left(u,v,w\right)$ be a triangle and p be an arbitrary point inside $T_{i}$. The number of relay nodes required to connect the vertices of $T_{i}$ at p is called the ap-weight of $T_{i}$ at p and it is denoted as $W_{ap}\left(T_{i},p\right)$. Basically, a relay node will be placed at p and then connected to the vertices u,v and w by placing the relay nodes on the edges $\bar{pu}$, $\bar{pv}$ and $\bar{pw}$, respectively. Thus,*

(1) $$\begin{array}{c} \begin{array}{cc} W_{ap}\left(T_{i},p\right) & =W_{e}\left(u,p\right)+W_{e}\left(v,p\right)+W_{e}\left(w,p\right)+1\\ & =\left(\left\lceil \frac{\left|up\right|}{R}\right\rceil -1\right)+\left(\left\lceil \frac{\left|vp\right|}{R}\right\rceil -1\right)+\left(\left\lceil \frac{\left|wp\right|}{R}\right\rceil -1\right)+1\\ & =\left\lceil \frac{\left|up\right|}{R}\right\rceil +\left\lceil \frac{\left|vp\right|}{R}\right\rceil +\left\lceil \frac{\left|wp\right|}{R}\right\rceil -2. \end{array} \end{array}$$

**Definition** **4.** *The point $s_{i}$ that minimizes the ap-weight of the triangle $T_{i}\left(u,v,w\right)$ is called the Steiner point and the weight at the Steiner point $s_{i}$ is called the sp-weight of $T_{i}$ and denoted as $W_{sp}\left(T_{i},s_{i}\right)$, or simply $W_{sp}\left(T_{i}\right)$. The Steiner point $s_{i}$ can be formally defined as follows:*

(2) $$\begin{array}{c} s_{i}=\min_{p}\left\{\left\lceil \frac{\left|up\right|}{R}\right\rceil +\left\lceil \frac{\left|vp\right|}{R}\right\rceil +\left\lceil \frac{\left|wp\right|}{R}\right\rceil -2\right\}. \end{array}$$

**Figure 2:**
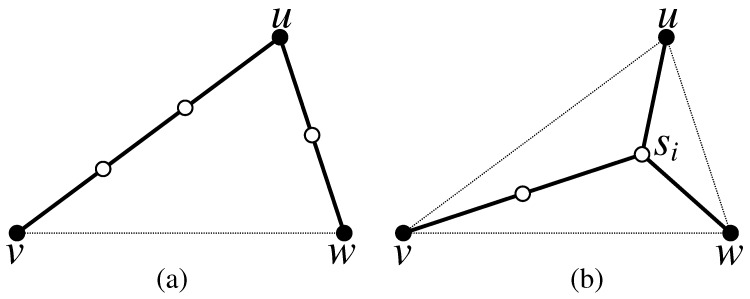
Illustration of two possible solutions for the problem of connecting tree terminals *u*, *v*, and *w* of a triangle $T_{i}=\left(u,v,w\right)$; (**a**) via Steinerizing the two smallest edges of $T_{i}$; and (**b**) via a Steiner point $s_{i}$. The solid nodes are terminals and hollow nodes are relay nodes.

If we multiply Equation (2) by “R” after removing the ceiling brackets, it follows that the minimization becomes linear in terms of distance. There are only two possible ways to achieve the optimal solution for connecting the three terminals *u*, *v*, and *w* of the triangle $T_{i}\left(u,v,w\right)$: (1) via Steinerizing the two smallest edges of $T_{i}$ as depicted in Figure 2a, which is equivalent to constructing the Steinerized MST for the three terminals; (2) via finding the Steiner point $s_{i}$ of $T_{i}$ and Steinerizing the edges $\bar{us_{i}}$, $\bar{vs_{i}}$ and $\bar{ws_{i}}$, as depicted in Figure 2b, where $∠us_{i}v=∠us_{i}w=∠vs_{i}w=120^{\circ }$.

**Theorem** **1.** The optimal solution for connecting three terminals u,v and w of a triangle $T_{i}\left(u,v,w\right)$ can be achieved either via Steinerizing the two smallest edges of $T_{i}\left(u,v,w\right)$ or Steinerizing the edges from its vertices to the Steiner point $s_{i}$.

**Proof.**  The proof of this theorem involves two cases:

Case 1: All relay nodes in the optimal solution lie on the edges joining the pairs of terminals. This case is straightforward and applicable only if there is no such point $p_{i}$ inside the triangle $T_{i}\left(u,v,w\right)$ that provides a better solution.

**Figure 3:**
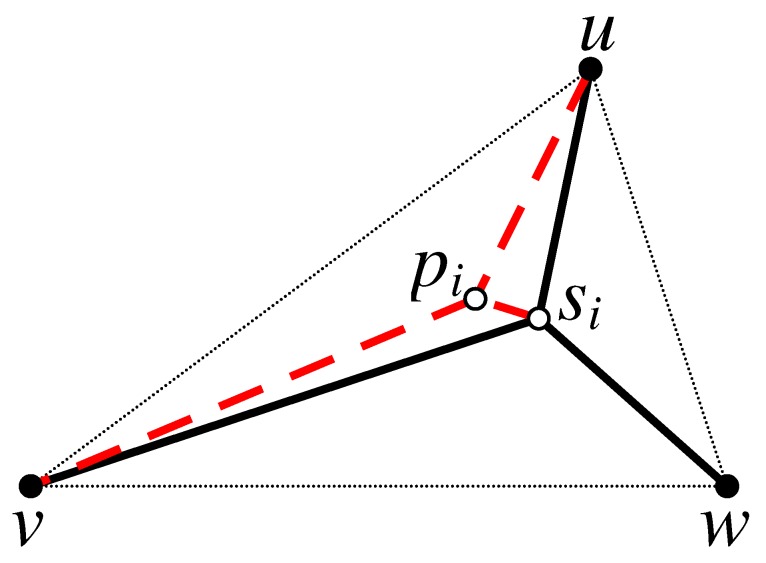
Illustrating how to connect the terminals *u*, $s_{i}$ and *v* of a sub-triangle $T_{i}^{\prime }=\left(u,s_{i},v\right)$ via an internal point $p_{i}$. The solid nodes are terminals and hollow nodes are relay nodes.

Case 2: There is a relay node inside the triangle $T_{i}\left(u,v,w\right)$ that connects all pairs of terminals in the optimal solution. From Definition 4, it is known that such a relay node is located at the Steiner point $s_{i}$ of $T_{i}\left(u,v,w\right)$. As a result of finding the Steiner point $s_{i}$ of the triangle $T_{i}\left(u,v,w\right)$, three sub-triangles namely $T_{i}^{\prime }\left(u,s_{i},v\right)$, $T_{i}^{\prime \prime }\left(u,s_{i},w\right)$ and $T_{i}^{\prime \prime \prime }\left(v,s_{i},w\right)$ are formed. In order to obtain a solution for connecting the terminals u,v and *w* of the triangle $T_{i}\left(u,v,w\right)$ via $s_{i}$, we first need to find the Steinerized MST for each of these sub-triangles. In order to prove the optimality, we need to show that the Steinerized MST is the optimal solution for the sub-triangles. We pursue our proof by way of contradiction. Assume that connecting the terminals of a sub-triangle, let say for instance $T_{i}^{\prime }\left(u,s_{i},v\right)$, via an internal point $p_{i}$ yields a better solution than Steinerizing the two smallest edges of $T_{i}^{\prime }\left(u,s_{i},v\right)$, as illustrated in Figure 3. Based on this assumption, we can write the following expressions:

$$\begin{array}{c} W_{ap}\left(T_{i}^{\prime },p_{i}\right)<W_{e}\left(u,s_{i}\right)+W_{e}\left(v,s_{i}\right), \end{array}$$

(3) $$\begin{array}{c} \left\lceil \frac{|s_{i}p_{i}|}{R}\right\rceil +\left\lceil \frac{|up_{i}|}{R}\right\rceil +\left\lceil \frac{|vp_{i}|}{R}\right\rceil -2<\left\lceil \frac{|us_{i}|}{R}\right\rceil +\left\lceil \frac{|vs_{i}|}{R}\right\rceil -2. \end{array}$$

Applying the triangular inequality on $T(w,s_{i},p_{i}),$ we get

$$\begin{array}{c} |ws_{i}\left|+\right|s_{i}p_{i}|\geq |wp_{i}|. \end{array}$$

Then,

$$\begin{array}{c} \frac{|ws_{i}|}{R}+\frac{|s_{i}p_{i}|}{R}\geq \frac{|wp_{i}|}{R}, \end{array}$$

and, hence,

(4) $$\begin{array}{c} \left\lceil \frac{|ws_{i}|}{R}\right\rceil +\left\lceil \frac{|s_{i}p_{i}|}{R}\right\rceil \geq \left\lceil \frac{|wp_{i}|}{R}\right\rceil . \end{array}$$

After connecting the terminals *u*, $s_{i}$ and *v* of the sub-triangle $T_{i}^{\prime }\left(u,s_{i},v\right)$ via an internal point $p_{i}$, the total number of relay nodes required for connecting the terminals *u*, *v* and *w* of the triangle $T_{i}\left(u,v,w\right)$ is the sum of e-weights of the edges $\bar{ws_{i}}$, $\bar{s_{i}p_{i}}$, $\bar{up_{i}}$, $\bar{vp_{i}}$ plus 2 (due to the two points $s_{i}$ and $p_{i}$). Therefore,

$$\begin{array}{c} \left(\left\lceil \frac{|ws_{i}|}{R}\right\rceil -1\right)+\left(\left\lceil \frac{|s_{i}p_{i}|}{R}\right\rceil -1\right)+\left(\left\lceil \frac{|up_{i}|}{R}\right\rceil -1\right)+\left(\left\lceil \frac{|vp_{i}|}{R}\right\rceil -1\right)+2 \end{array}$$

$$\begin{array}{c} =\left\lceil \frac{|ws_{i}|}{R}\right\rceil +\left\lceil \frac{|s_{i}p_{i}|}{R}\right\rceil +\left\lceil \frac{|up_{i}|}{R}\right\rceil +\left\lceil \frac{|vp_{i}|}{R}\right\rceil -2. \end{array}$$

Using Equation (4), we get

(5) $$\begin{array}{c} \left\lceil \frac{|ws_{i}|}{R}\right\rceil +\left\lceil \frac{|s_{i}p_{i}|}{R}\right\rceil +\left\lceil \frac{|up_{i}|}{R}\right\rceil +\left\lceil \frac{|vp_{i}|}{R}\right\rceil -2\geq \left\lceil \frac{|wp_{i}|}{R}\right\rceil +\left\lceil \frac{|up_{i}|}{R}\right\rceil +\left\lceil \frac{|vp_{i}|}{R}\right\rceil -2. \end{array}$$

Based on Definition 3, $\left\lceil \frac{|wp_{i}|}{R}\right\rceil +\left\lceil \frac{|up_{i}|}{R}\right\rceil +\left\lceil \frac{|vp_{i}|}{R}\right\rceil -2$ is the ap-weight of the triangle $T_{i}\left(u,v,w\right)$ at point $p_{i}$, and it is denoted as $W_{ap}\left(T_{i},p_{i}\right)$. Consequently, Equation (5) becomes

(6) $$\begin{array}{c} \left\lceil \frac{|ws_{i}|}{R}\right\rceil +\left\lceil \frac{|s_{i}p_{i}|}{R}\right\rceil +\left\lceil \frac{|up_{i}|}{R}\right\rceil +\left\lceil \frac{|vp_{i}|}{R}\right\rceil -2\geq W_{ap}\left(T_{i},p_{i}\right). \end{array}$$

Using Equation (3), we can write

(7) $$\begin{array}{c} W_{sp}\left(T_{i}\right)=\left\lceil \frac{|us_{i}|}{R}\right\rceil +\left\lceil \frac{|vs_{i}|}{R}\right\rceil +\left\lceil \frac{|ws_{i}|}{R}\right\rceil -2>\left\lceil \frac{|ws_{i}|}{R}\right\rceil +\left\lceil \frac{|s_{i}p_{i}|}{R}\right\rceil +\left\lceil \frac{|up_{i}|}{R}\right\rceil +\left\lceil \frac{|vp_{i}|}{R}\right\rceil -2. \end{array}$$

From Equations (6) and (7), we have $W_{sp}\left(T_{i}\right)>W_{ap}\left(T_{i},p_{i}\right)$. However, this is a contradiction since the definition of a Steiner point $s_{i}$ implies that $W_{sp}\left(T_{i}\right)$ is minimal and cannot be greater than $W_{ap}\left(T_{i},p_{i}\right)$. Therefore, $p_{i}$ of $T_{i}^{\prime }\left(u,s_{i},v\right)$ must be equal to $s_{i}$, which is equivalent to constructing the Steinerized MST for the sub-triangle $T_{i}^{\prime }\left(u,s_{i},v\right)$. The same proof can be applied to $T_{i}^{\prime \prime }\left(u,s_{i},w\right)$ and $T_{i}^{\prime \prime \prime }\left(w,s_{i},v\right)$. To complete our proof, it is worth noting that if we simultaneously consider Steinerizing two of the three sub-triangles via the Steiner point $s_{i}$, the resulting topology will not be cycle-free. Thus, we can conclude that considering two (or three) sub-triangles simultaneously will increase the required number of relay nodes and hence provide no benefit to the solution. ☐

Theorem 1 suggests that RPSNC optimally solves the problem of re-establishing the network connectivity for the case of three terminals. It is challenging to design an optimal algorithm for re-establishing the network connectivity for larger networks because, as we mentioned earlier, the problem is NP-hard.

**Figure 4:**
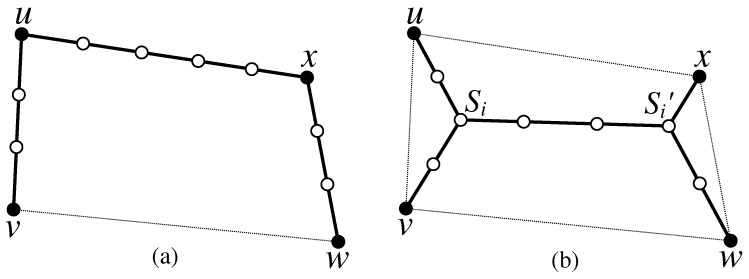
Illustration of two possible solutions for the problem of connecting four terminals *u*, *v*, *w* and *x* of a quadrilateral $Q_{i}=\left(u,v,w,x\right)$; (**a**) via Steinerizing the three smallest edges of $Q_{i}$; and (**b**) via two Steiner points $s_{i}$ and $s_{i}^{\prime }$. The solid nodes are terminals and hollow nodes are relay nodes.

**Definition** **5.** Let $Q_{i}\left(u,v,w,x\right)$ be a quadrilateral. There are two possible solutions for connecting the four terminals u, v, w and x of $Q_{i}$: (1) via Steinerizing the three smallest edges of $Q_{i}$ as depicted in Figure 4a, which is equivalent to constructing the Steinerized MST for the four terminals; (2) via finding the Steiner points $s_{i}$ and $s_{i}^{\prime }$ of $Q_{i}$ and Steinerizing the edges $\bar{us_{i}}$, $\bar{vs_{i}}$, $\bar{s_{i}s_{i}^{\prime }}$, $\bar{xs_{i}^{\prime }}$ and $\bar{ws_{i}^{\prime }}$, as depicted in Figure 4b, where $∠us_{i}v=∠vs_{i}s_{i}^{\prime }=∠xs_{i}^{\prime }w=∠ws_{i}^{\prime }s_{i}=120^{\circ }$.

**Definition** **6.** *The number of relay nodes required for connecting the terminals of a quadrilateral $Q_{i}=\left(u,v,w,x\right)$ by forming MST of these terminals, as depicted in Figure 4a, is called the MST-Weight of $Q_{i}$, denoted as $W_{MST}\left(Q_{i}\right)$, and computed as*

$$\begin{array}{c} W_{MST}\left(Q_{i}\right)=\left\lceil \frac{\left|uv\right|}{R}\right\rceil +\left\lceil \frac{\left|xu\right|}{R}\right\rceil +\left\lceil \frac{\left|wx\right|}{R}\right\rceil -3, \end{array}$$

*where R is the communication range of a relay node.*

**Definition** **7.** *The number of relay nodes required for connecting the terminals of a quadrilateral $Q_{i}=\left(u,v,w,x\right)$ via the Steiner points $s_{i}$ and $s_{i}^{\prime }$, as illustrated in Figure 4b is called the C-weight of $Q_{i}$, denoted as $W_{c}\left(Q_{i}\right)$ and computed as*

$$\begin{array}{c} W_{c}\left(Q_{i}\right)=\left\lceil \frac{|s_{i}u|}{R}\right\rceil +\left\lceil \frac{|s_{i}v|}{R}\right\rceil +\left\lceil \frac{|s_{i}^{\prime }x|}{R}\right\rceil +\left\lceil \frac{|s_{i}^{\prime }w|}{R}\right\rceil +\left\lceil \frac{|s_{i}s_{i}^{\prime }|}{R}\right\rceil -3. \end{array}$$

## 4. A Heuristic RPSNC Algorithm for Establishing Inter-Partitions Connectivity

### 4.1. The Main Idea of RPSNC

The main idea of the proposed RPSNC is to optimize the number and positions of required relay nodes for restoring inter-partitions connectivity for N≥3 given disjoint partitions of a WSN topology, assuming intra-partition connectivity. RPSNC uses linear programming as a means of optimization for minimizing the required number of relay nodes. RPSNC combines non-uniform partitioning and Delaunay triangulation techniques for generating the candidate relay nodes, after which, linear programming is applied for choosing the optimal relay nodes and determining their connection links with the terminals. Subsequently, an equilibrium method is used to refine the locations of the chosen relay nodes, by moving them to balanced positions. RPSNC connects all partitions at once, which may enable us to further reduce the required number of relay nodes.

### 4.2. Detailed Description of RPSNC

RPSNC consists of two phases: Phase I aims to use non-uniform partitioning and Delaunay triangulation techniques for generating the candidate relay nodes. It also determines the *topology* of the restored WSN by using linear programming. Phase II aims to determine the optimal positions of the relay nodes in the *topology* obtained in Phase I by using an equilibrium method.

#### 4.2.1. Phase I: Computing the Topology of the Restored WSN

The following two scenarios need to be handled for generating the candidate relay nodes: non-uniform density distribution of terminals; and non-uniform density distribution between relay nodes and terminals. To achieve this, we combine non-uniform partitioning and Delaunay triangulation techniques.

**Figure 5:**
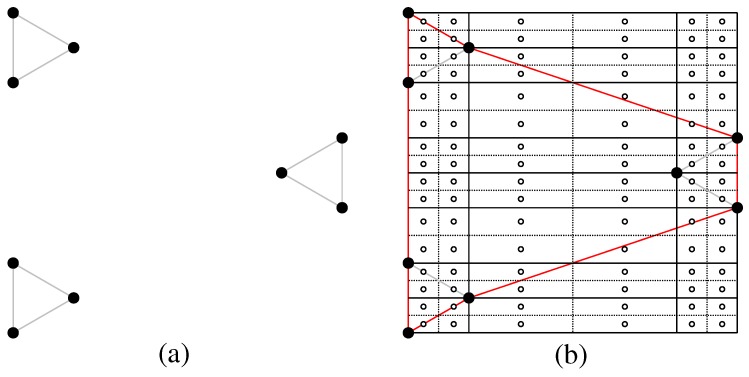
Example of non-uniform partitioning. (**a**) Nine given clustering terminals in 2D Euclidean space; (**b**) non-uniform partitioning: the solid nodes are terminals and hollow nodes are relay nodes.

Non-uniform partitioning can handle any density distribution of terminals, particularly non-uniform distributions. As example, consider nine clustering terminals depicted in Figure 5a. Figure 5b illustrates the non-uniform partitioning. First, compute a convex hull (in red) for these nine terminals. Second, draw a vertical line and a horizontal line through every terminal to obtain a bounding box and a number of sub-rectangles of different sizes. Third, partition recursively every sub-rectangle into q×q cells, where *q* denotes the non-uniform partitioning parameter, which is a positive integer. Finally, the centers of cells inside the convex hull are taken as the candidate relay nodes. With non-uniform partitioning, the distribution of the candidate relay nodes is in accordance with the terminals, which can speedup the convergence of RPSNC because the candidate relay nodes outside the convex hull are not considered.

RPSNC adopts the following linear programming model:

**Minimize** $\;cost=\sum_{\vec{uv}\in A}w\left(\vec{uv}\right)f\left(\vec{uv}\right)$

**Subject to :**

(8) $$\begin{array}{c} \left\{\begin{array}{cc} \sum_{v\in V_{↑}\left(u\right)}f_{i}\left(\vec{vu}\right)=\sum_{v\in V_{↓}\left(u\right)}f_{i}\left(\vec{uv}\right) & \forall i,\forall u\\ f_{i}\left(\vec{T_{i}S}\right)=r & \forall i\\ f_{i}\left(\vec{uv}\right)\leq f\left(\vec{uv}\right) & \forall i,\forall \vec{uv}\\ f\left(\vec{uv}\right)\geq 0,f_{i}\left(\vec{uv}\right)\geq 0 & \forall i,\forall \vec{uv}. \end{array}\right. \end{array}$$

The linear programming model (Equation (8)) is based on a directed network G=(V,A), where *V* is the set of *N* terminal nodes and *M* additional relay nodes, while $A=\{\vec{uv},\vec{vu}|uv\in V\}$ is the set of directed links. In the linear programming objective function, the decision variable $f(\vec{uv})$ represents the combined effective flow rate on a link $\vec{uv}\in A$. The coefficient (i.e., weight) $w(\vec{uv})$ is the Euclidean distance $|\vec{uv}|$(=$|\vec{vu}|$=|uv|) of the link $\vec{uv}$. In the linear programming constraints, $f_{i}\left(\vec{uv}\right)$ is regarded as the rate of information flow from the source node *S* to the receiving node $T_{i}$ on a link $\vec{uv}$. For every network information flow S→$T_{i}$, there is a *conceptual* flow $f_{i}\left(uv\right)$ [32]. We call it *conceptual* because different conceptual flows share instead of competing for available bandwidth on the same link [32]. The final flow rate $f(\vec{uv})$ of a link uv equals to the maximum among all $f_{i}\left(\vec{uv}\right)$ and should be not less than the maximum conceptual rate, which will directly affect the total cost. $V_{↑}\left(u\right)$ and $V_{↓}\left(u\right)$ denote upstream and downstream adjacent set of *u* in *V*, respectively. *r* is a multicast rate from the source *S* to each sink $T_{i}$ and it is set to 1. We assume that there is a conceptual link from each sink $T_{i}$ back to the source *S* with the rate *r*, for concise representation of flow conservation constraints [32]. For every pair of nodes, we have both $f_{i}\left(\vec{uv}\right)$ and $f_{i}\left(\vec{vu}\right)$ to indicate the flows in two directions.

As denoted by Huang et al. [14], non-uniform partitioning suffers from the approaching-infinity problem, i.e., when the number of candidate relay nodes approaches infinity, the number of decision variables of linear programming model (Equation (8)) approaches infinity too, which makes the algorithm adopting the linear programming model (Equation (8)) not terminate in polynomial time.

To solve the approaching-infinity problem, we adopt the Delaunay triangulation technique. Delaunay triangulation is a computational geometric technique that produces a superset of MST. Since every Delaunay triangle tends to be approximately equilateral, we can achieve the maximum possible reduction of length. Delaunay triangulation has been used to solve the problem of ESMT [31] and hence we can use it to solve the problem of SIF since SIF consists of minimum superposition of ESMT [14]. This work proposes a Delaunay triangulation based technique (Lines 8–10 of Algorithm 1) that is explained as follows. First, use a Delaunay triangulation algorithm [31] to generate Delaunay triangles for N≥3 given terminals (Line 8). Second, generate the candidate Steiner nodes for each Delaunay triangle (Line 9), which is equivalent to three-terminals ESMT problem and can be computed in polynomial time by the Simpson method [16]. Third, generate the candidate Steiner nodes for each quadrilateral obtained by concatenating two adjacent Delaunay triangles (Line 10), which is equivalent to four-terminals ESMT problem and can be computed in polynomial time by the iterative equilateral point method [16]. It is not necessary to compute the candidate Steiner nodes for 5, 6, … *N* terminals (*N*-terminals ESMT problem), since the optimal solution (with regard to a certain *q*) can be obtained by the second computation of linear programming model (Line 20 of Algorithm 1), as it will be verified later by simulation experiments.

#### 4.2.2. Finding the Optimal Positions of the Relay Nodes

**Figure 6:**
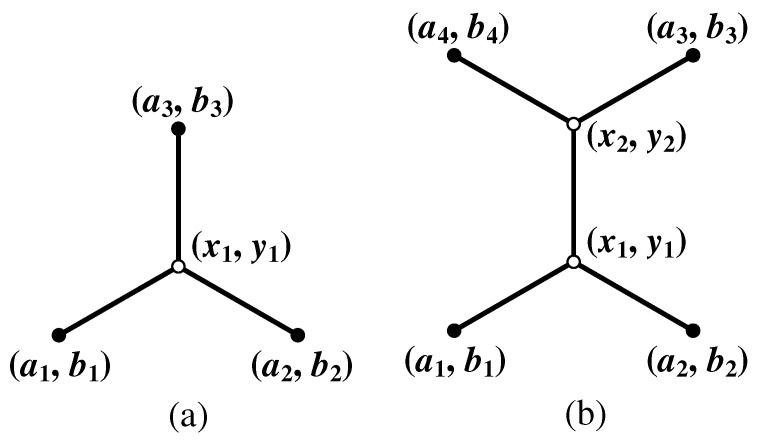
The analytic geometric method for equilibrium in Phase II. (**a**) one relay node with three adjacent terminals; (**b**) two relay nodes that are adjacently connected and four adjacent terminals.

The goal of equilibrium method (Line 18 of Algorithm 1) is to fine-tune the relay nodes chosen by linear programming towards their optimal positions, which satisfy the balanced [33] property of optimal SIF stable at relay. Since each relay node chosen by linear programming satisfies the $120^{\circ }$ property [14], we adopt the analytic geometric method [34] for equilibrium. The analytic geometric method is based on the $120^{\circ }$ condition: if a relay node has three adjacent links each with equal flow rate, then the balanced position of the relay node should result in three $120^{\circ }$ angles among its three adjacent links. The analytic geometric method exploits this fact to compute the coordinates of the balanced relay nodes. More specifically, we can apply *inner product* of two vectors to establish equations. Let us suppose that a relay node ($x_{1}$, $y_{1}$) is connected with three adjacent terminals ($a_{1},b_{1}$), ($a_{2},b_{2}$) and ($a_{3},b_{3}$) (see Figure 6a). The following two equations can be solved to get the two unknown variables (i.e., $x_{1}$ and $y_{1}$):

(9) $$\begin{array}{c} \left\{\begin{array}{c} \frac{\left(a_{1}-x_{1}\right)\left(a_{2}-x_{1}\right)+\left(b_{1}-y_{1}\right)\left(b_{2}-y_{1}\right)}{\sqrt{{\left(a_{1}-x_{1}\right)}^{2}+{\left(b_{1}-y_{1}\right)}^{2}}\sqrt{{\left(a_{2}-x_{1}\right)}^{2}+{\left(b_{2}-y_{1}\right)}^{2}}}=\cos\;120^{\circ }\\ \frac{\left(a_{1}-x_{1}\right)\left(a_{3}-x_{1}\right)+\left(b_{1}-y_{1}\right)\left(b_{3}-y_{1}\right)}{\sqrt{{\left(a_{1}-x_{1}\right)}^{2}+{\left(b_{1}-y_{1}\right)}^{2}}\sqrt{{\left(a_{3}-x_{1}\right)}^{2}+{\left(b_{3}-y_{1}\right)}^{2}}}=\cos\;120^{\circ }. \end{array}\right. \end{array}$$

The number of equations varies according to the following cases. If a relay node ($x_{1}$, $y_{1}$) has one adjacent relay node ($x_{2}$, $y_{2}$) and two adjacent terminals ($a_{1}$, $b_{1}$) and ($a_{2}$, $b_{2}$), there should be four equations with four unknown variables, as $x_{2}$ and $y_{2}$ have another two equations. Suppose that the relay node ($x_{2}$, $y_{2}$) has two adjacent terminals ($a_{3}$, $b_{3}$) and ($a_{4}$, $b_{4}$) (see Figure 6b). The following four equations can be solved to get the four unknown variables (i.e., $x_{1}$, $y_{1}$, $x_{2}$ and $y_{2}$):

(10) $$\begin{array}{c} \left\{\begin{array}{c} \frac{\left(a_{1}-x_{1}\right)\left(a_{2}-x_{1}\right)+\left(b_{1}-y_{1}\right)\left(b_{2}-y_{1}\right)}{\sqrt{{\left(a_{1}-x_{1}\right)}^{2}+{\left(b_{1}-y_{1}\right)}^{2}}\sqrt{{\left(a_{2}-x_{1}\right)}^{2}+{\left(b_{2}-y_{1}\right)}^{2}}}=\cos\;120^{\circ }\\ \frac{\left(a_{1}-x_{1}\right)\left(x_{2}-x_{1}\right)+\left(b_{1}-y_{1}\right)\left(y_{2}-y_{1}\right)}{\sqrt{{\left(a_{1}-x_{1}\right)}^{2}+{\left(b_{1}-y_{1}\right)}^{2}}\sqrt{{\left(x_{2}-x_{1}\right)}^{2}+{\left(y_{2}-y_{1}\right)}^{2}}}=\cos\;120^{\circ }\\ \left(a_{1}-x_{1}\right)/\left(b_{1}-y_{1}\right)=\left(a_{3}-x_{2}\right)/\left(b_{3}-y_{2}\right)\\ \left(a_{2}-x_{1}\right)/\left(b_{2}-y_{1}\right)=\left(a_{4}-x_{2}\right)/\left(b_{4}-y_{2}\right). \end{array}\right. \end{array}$$

The latter two equations come from the two parallel vectors. For example, the last equation is due to two parallel vectors ($a_{2}$ – $x_{1}$, $b_{2}$ – $y_{1}$) and ($a_{4}$ – $x_{2}$, $b_{4}$ – $y_{2}$). This can reduce the computation overhead. If a relay node has two adjacent relay nodes and one adjacent terminal, there should be six equations with six unknown variables. For each extra relay node (two unknown coordinates), two extra equations arise. The number of unknown variables is always equal to the number of equations.

**Algorithm 1** RPSNC Algorithm**Require:** Input: a partitioned WSN topology**Ensure:** Output: a restored connectivity WSN topology1:Initialization: MINCOST${}_{I}$ = MINCOST${}_{II}$ = +*∞*, partitioning coefficient *q* = 2;2:Mark each partition as a disconnected partition;3:Select one representative sensor node in each partition and set it as a *terminal*;4:**for**
N≥3 terminals from all partitions, **do**5: Compute a convex hull for *N* terminals;6: Execute the non-uniform partitioning with *q*;7: Consider the centers that are inside the convex hull as the candidate relay nodes;8: Construct all Delaunay triangles for *N* terminals by Delaunay triangulation;9: Generate the candidate Steiner nodes for every Delaunay triangle;10: Generate the candidate Steiner nodes for every quadrilateral obtained by concatenating two adjacent Delaunay triangles;11: Add the candidate Steiner nodes to the set of the candidate relay nodes;12:**end for**13:Construct a complete graph with *N* terminals and the candidate relay nodes of the *current* round as well as the *balanced relay nodes* of the *last* round;14:Solve the linear programming model (Equation (8)) based on the complete graph and output the *resulting relay nodes*;15:**if**
$cost_{q}<$ MINCOST${}_{I}$
**then**16: MINCOST${}_{I}$ = $cost_{q}$;17:**end if**18:Apply the analytic geometric method for equilibrium to get the exact coordinates of the *balanced relay nodes*;19:Construct the second complete graph with *N* terminals and the balanced relay nodes;20:Solve the linear programming model (Equation (8)) based on the second complete graph;21:**if**
$cost_{q}<$ MINCOST${}_{II}$
**then**22: MINCOST${}_{II}$ = $cost_{q}$;23:**end if**24:**if** MINCOST${}_{I}$≠ MINCOST${}_{II}$
**then**25: Goto Step 13;26:**end if**27:**if** MINCOST${}_{II}$ of current round ≠ MINCOST${}_{II}$ of last round **then**28: q=q+1 and goto Step 6;29:**else**30: Output MINCOST${}_{II}$ and stop.31:**end if**32:**if** Resulting *balanced relay nodes* cover all terminals **then**33: Output a connectivity restored WSN topology and stop.34:**else**35: Deploy additional relay nodes along the connection links such that all the terminals are covered and stop.36:**end if**

The pseudo code of RPSNC is shown in Algorithm 1. Two linear programming computations are adopted by Line 14 and Line 20 before and after the equilibrium method, respectively. The linear programming computation before equilibrium aims to obtain the topology, while the one after equilibrium can help to decide when to stop RPSNC. The two linear programming models are the same, but their input complete graphs are different. The balanced relay nodes that are obtained by equilibrium method of Phase II (Line 18 of Algorithm 1) represent local optimization to some extent. Therefore, a retention mechanism [34] that can speedup the convergence is adopted by Line 13. In addition, the value of MINCOST${}_{II}$ will decrease monotonically, which can also help to decide when RPSNC can stop.

### 4.3. Illustrative Example

**Figure 7:**
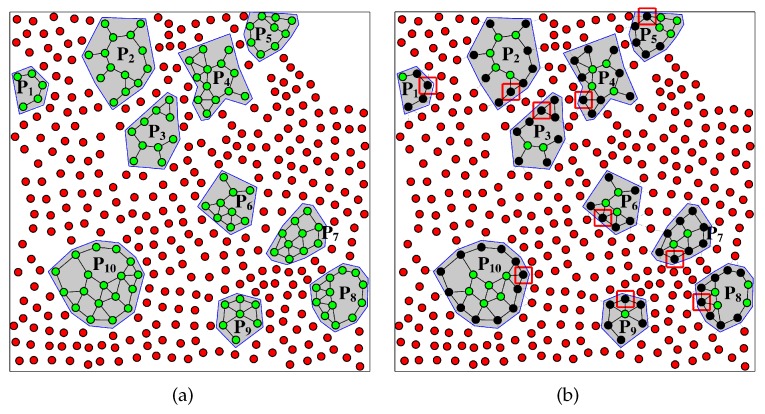
(**a**) A partitioned WSN due to large scale damage. Green nodes represent operational nodes, while red nodes represent failed nodes; (**b**) selection of partitions representative nodes. Black nodes marked by a square are representative nodes (terminals) of each partition selected among black (border) nodes that detect the damage.

**Figure 8:**
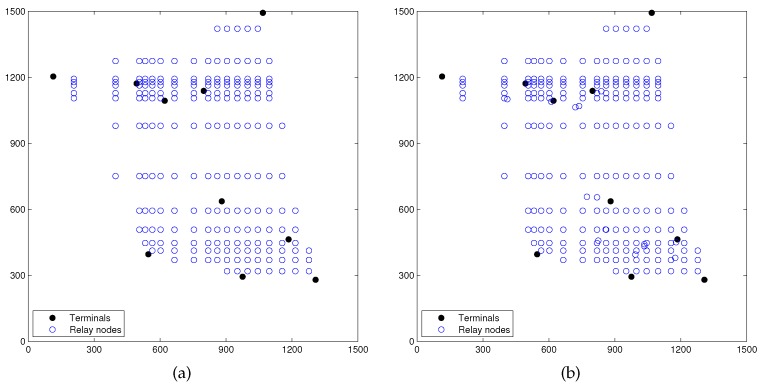
(**a**) terminals and candidate relay nodes from non-uniform partitioning (q = 2) of Phase I of RPSNC for random network (N=10); (**b**) terminals and candidate relay nodes from non-uniform partitioning (q = 2) and Delaunay triangulation of Phase I of RPSNC.

**Figure 9:**
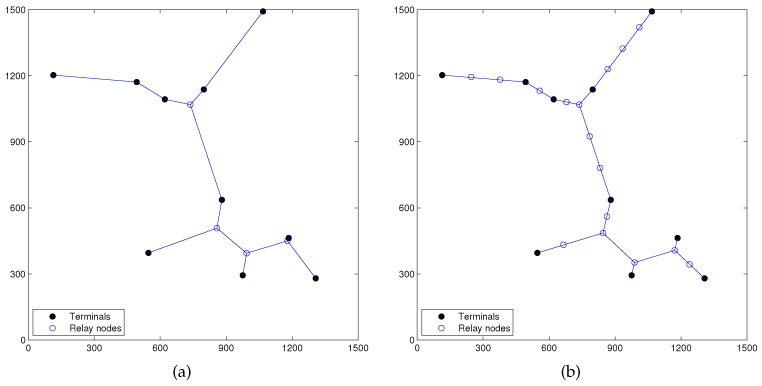
(**a**) Topology obtained from the first round of linear programming computation of Phase I of RPSNC (min-cost = 2804.586319/bit); (**b**) final topology by RPSNC after the second round of linear programming computation of Phase II, R = 100 m (min-cost = 2800.183681/bit).

Figure 7, Figure 8 and Figure 9 illustrate how RPSNC works through a detailed example of random network. Let us assume that we have a partitioned WSN deployed in 1500 m × 1500 m area with ten partitions, as illustrated in Figure 7a. In order to restore the connectivity among partitions of a damaged WSN, RPSNC first selects a representative sensor node $REP(P_{i})$ for each partition $P_{i}$ (Line 3 of Algorithm 1) by CORP algorithm [4]. We assume that only one representative sensor node is selected for each partition. In order to decide the representative sensor nodes for the case of reconnecting a set of partitions in a damaged WSN, it is required to first address one major issue: how the surviving sensor nodes recognize that a network is split into partitions.

Since the partition is due to a major scale damage inflicted by uncontrolled event/force such as explosions, the closest sensor nodes to the affected area—for example, black sensor nodes in Figure 7b detect the failure of their neighbors. For instance, they may conclude that there is major damage when they detect consecutive node failures, when they notice a huge and sudden drop in communication traffic and/or when they become unable to reach a certain set of sensor nodes. Upon confirming the damage, the black nodes send a message on active links to notify all reachable sensor nodes. The damage-detection message is delivered to all sensor nodes that belong to the same partition.

After some pre-determined convergence time, the black sensor node that has more neighbors than other border (black) sensor nodes within the same partition becomes a representative sensor node. This can be tracked by including the number of neighbors in the notification messages that get flooded in the partition. The rationale of the REP() selection is that new relay nodes are deployed in the vicinity of these border sensor nodes, and it is thus imperative to restore the network topology in a manner as similar as possible to its pre-failure state. In Figure 7b, a black node in a square becomes a $REP(P_{i})$ for each partition $P_{i}$. Therefore, ten REP()s are selected for ten partitions in Figure 7b, and their locations are announced according to the other sensor nodes in the partitions. The problem of federating the ten partitions shown in Figure 7a is now equivalent to reconnecting $REP(P_{i})$, i=1,2,3,…10. These ten $REP(P_{i})$ are set as terminals.

Figure 8a shows the terminals and candidate relay nodes from non-uniform partitioning of Phase I of RPSNC. Figure 8b shows the terminals and candidate relay nodes from non-uniform partitioning, as well as the candidate relay nodes from Delaunay triangulation of Phase I of RPSNC. Figure 9a shows the topology obtained from the first round of linear programming computation of Phase I of RPSNC, with a min-cost of 2804.586319/bit. Figure 9b shows the final topology by RPSNC, which is obtained after the second round of linear programming computation of Phase II, with a min-cost of 2800.183681/bit. Figure 9b shows that the total 16 relay nodes are used in the recovery process when R = 100 m.

### 4.4. Analysis of RPSNC

In this subsection, we analyze the performance and complexity of the proposed RPSNC. We prove that RPSNC always outperforms (or equal to in the worst case) FeSTA and QTA, and it has a polynomial complexity.

#### 4.4.1. Performance Analysis

We introduce the following theorem for analyzing the performance of RPSNC:

**Theorem** **2.** RPSNC always performs better than (or equal to in the worst case) FeSTA and QTA.

**Proof.**  Both FeSTA and QTA aim to improve the MST-based solution by leveraging the Steiner method based Steinerization. Let $T_{i}\left(u,v,w\right)$ be a triangle, if (u,v) and (v,w) are Steinerized edges of $T_{i}$, the optimization gain of $T_{i}$ can be calculated as $Gain\left(T_{i}\right)=W_{e}\left(u,v\right)+W_{e}\left(v,w\right)-W_{sp}\left(T_{i}\right)$. FeSTA lists N3=$\frac{N^{3}-3N^{2}+2N}{6}$ possible triangles, among which those having positive gain are considered for optimization. On the other hand, QTA lists N4=$\frac{N^{4}-6N^{3}+11N^{2}-6N}{24}$ possible quadrilaterals, among which convex quadrilaterals are considered for optimization. In the worst case where no triangles and quadrilaterals are found to be optimized by Steiner method based Steinerization, both FeSTA and QTA end up with the Steinerized MST. In other words, running either FeSTA or QTA never produces a topology that requires more relay nodes than MST-based solution. While FeSTA and QTA only consider triangles and quadrilaterals Steiner nodes, respectively, RPSNC takes full advantage of considering Delaunay triangles and quadrilaterals Steiner nodes (Lines 9–10 of Algorithm 1). Thus, RPSNC is hybrid to FeSTA and QTA to a certain extent. RPSNC uses linear programming as a means of optimization to choose the minimum number of required relay nodes. The following three cases arise: (1) if linear programming chooses the relay nodes from Delaunay triangles and quadrilaterals, then RPSNC results will be better than those of FeSTA and QTA. (2) If linear programming chooses the relay nodes from Delaunay triangles only, then RPSNC results will be equal to FeSTA results. (3) If linear programming chooses the relay nodes from quadrilaterals only, then RPSNC results will match those of QTA. Therefore, RPSNC will always perform better than (or equal to) FeSTA and QTA. ☐

Theorem 2 suggests that RPSNC significantly performs better than FeSTA and QTA.

#### 4.4.2. Complexity Analysis

The time complexity of selecting the partitions representative sensor nodes (Line 3 of Algorithm 1) is O(r.N) [4], where *r* is the number of rounds. On Line 6, the upper-bound number of the candidate relay nodes from non-uniform partitioning is $q^{2}{\left(N-1\right)}^{2}$. The time complexity for constructing Delaunay triangles (Line 8) is O(NlogN) [31]; the time complexity of computing all the candidate Steiner nodes for every Delaunay triangle (Line 9) is O(2N-5) since we can get at most 2N-5 Delaunay triangles for *N* terminals; the time complexity of computing all the candidate Steiner nodes for every quadrilateral obtained by concatenating two adjacent Delaunay triangles (Line 10) is O(3N-6) because we can get at most 3N-6 Delaunay triangulation edges for *N* terminals; the upper-bound number of the candidate Steiner nodes for all Delaunay triangles is 2N-5 since each Delaunay triangle has at most one candidate Steiner node; the upper-bound number of the candidate Steiner nodes from quadrilaterals formed by concatenating all two adjacent Delaunay triangles is 2(3N-6) because each quadrilateral has at most two Steiner nodes. Line 13 deals with constructing a complete graph (CG) for all the nodes and $N_{CG}=N+q^{2}{\left(N-1\right)}^{2}+\left(2N-5\right)+2\left(3N-6\right)$, where $N_{CG}$ denotes the number of nodes in the complete graph. The number of linear programming decision variables is the number of edges of the complete graph, i.e., NCG2, whose time complexity is $O(q^{4}N^{4})$. The time complexity of linear programming constraints is $O(q^{4}N^{5})$. Since the combination of Delaunay triangulation and non-uniform partitioning techniques eliminates the approaching-infinity problem, the partitioning parameter *q* can be a finite constant that is independent from *N*. Hence, the time complexity of linear programming at Line 14 is polynomial. The time complexity of analytic geometric method (Line 18) is polynomial because it can compute the exact coordinates of the relay nodes by solving Equations (9) and (10), whose upper-bound number is N-2. The iterations of the main loop of RPSNC is q-1. In summary, the time complexity of RPSNC is polynomial. As for space complexity, the amount of storage required for constructing Delaunay triangles is O(N) [31]. Since there are $q^{2}{\left(N-1\right)}^{2}+\left(2N-5\right)+2\left(3N-6\right)$ relay nodes, the amount of storage required for them is $O(q^{2}N^{2})$. The amount of storage required for linear programming decision variables is $O(q^{4}N^{4})$ since there are NCG2 edges in the complete graph. The amount of storage required for linear programming constraints is $O(q^{4}N^{5})$. The partitioning parameter *q* is a finite constant that is independent from *N*. Therefore, the space complexity of linear programming at line 14 is polynomial. In summary, the space complexity of RPSNC is also polynomial.

## 5. Performance Evaluation

### Experiment Setup and Performance Metrics

In the simulation, two sets of experiments have been conducted in 1500 m × 1500 m area for random networks and Pentagram [15] network. In the first set, the number of partitions is varied from 4 to 10 while fixing the communication range *R* at 100 m. In the second set, the number of partitions is fixed to 10 for random networks and 6 for Pentagram network, and random topologies are created with varying *R* from 50 m to 200 m, while the Pentagram network is created with varying *R* from 50 m to 350 m. The second set of experiments is geared for studying the impact of *R*. The Pentagram network is applied to the second set of experiments only because the number of partitions can not be varied in Pentagram network. Our simulations used MATLAB R2012b to solve linear programming model (Equation (8)). The performance of RPSNC is compared to three baseline approaches, namely, FeSTA [3], QTA [6] and MST_1TRNP [9]. The following metrics are considered for evaluating the performance: 

**Number of relay nodes**: This metric shows the total number of relay nodes required to restore the connectivity. Obviously, minimizing the required relay node count is the objective of the optimization and for sure captures the effectiveness of RPSNC.

**Average node degree**: This metric reports the average number of neighbors for each node in the resulting topology. A higher node degree indicates a stronger connectivity and enables better load balancing among the routing resources, which reduces the data latency.

#### 5.1.1. Random Networks

**Figure 10:**
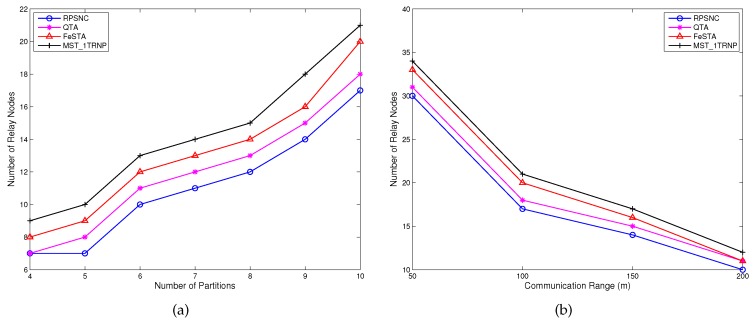
(**a**) Comparing the relay nodes count performance of RPSNC to the baseline approaches, under varying the number of partitions in random networks; (**b**) comparing the relay nodes count performance of RPSNC to the baseline approaches, with varying *R* in random networks.

**Number of relay nodes**: Figure 10a shows that RPSNC performs significantly better than QTA, FeSTA and MST_1TRNP in terms of the number of relay nodes required to restore the connectivity, under varying the number of partitions. The reason of such performance can be explained as follows. Unlike FeSTA and QTA that are based on triangle Steiner tree and quadrilateral Steiner tree methods, respectively, RPSNC takes full advantage of combining both methods. In contrast to MST_1TRNP that deploys the relay nodes along the MST edges, RPSNC places them at the Steiner points inside the Delaunay triangles and quadrilaterals, which minimizes the required relay node count. It is worth noting that, for all algorithms, the number of relay nodes increases when the number of partitions increases due to the fact that more relay nodes are required to connect more partitions. Figure 10b shows the effect of *R* on relay node count. For bigger transmission ranges, all algorithms restore the connectivity with fewer relay nodes, which is intuitive. RPSNC again outperforms QTA, FeSTA and MST_1TRNP.

**Figure 11:**
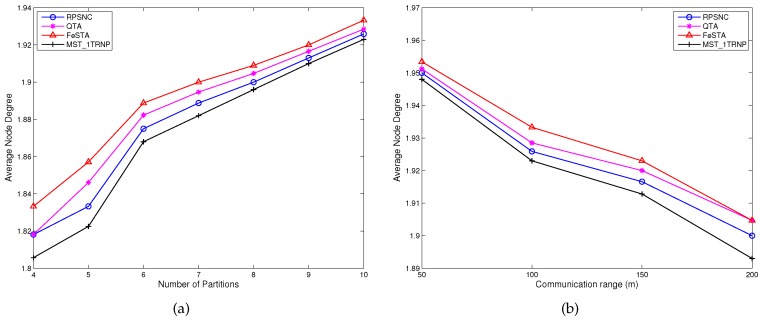
(**a**) The average node degree of RPSNC and the baseline approaches, under varying the number partitions in random networks; (**b**) the average node degree of RPSNC and the baseline approaches, with varying *R* in random networks.

**Average node degree**: Figure 11a,b reports the average node degree for all compared approaches. Figure 11a compares the performance in terms of average node degree under varying the number of partitions. The average node degree of all compared algorithms increases as the number of partitions increases. This can be attributed to the fact that more relay nodes are required to connect more partitions, which increases the average node degree. RPSNC requires fewer relay nodes and yields stronger connectivity than MST_1TRNP. This can be attributed to the fact that every relay node in MST_1TRNP has a node degree of two because all relay nodes are deployed along the edges of MST, while RPSNC places some of the relay nodes at the Steiner points of the Delaunay triangles and quadrilaterals, yielding a node degree of three and consequently a strong connectivity. Therefore, if there are more Delaunay triangles and quadrilaterals to be optimized by RPSNC, the average node degree gets closer to three because more relay nodes will have a node degree of three. Interestingly, however, QTA and FeSTA yield higher average node degree than RPSNC, since they require more relay nodes than RPSNC and place some of the relay nodes to the Steiner points of the triangles and quadrilaterals, just like RPSNC does. Figure 11b compares the performance in terms of average node degree under varying the communication range of the relay nodes (*R*). Interestingly, the average node degree of all compared algorithms decreases as the transmission range increases. The reason is that when the transmission range of the relay nodes increases, the number of relay nodes required reduces, which decreases the average node degree. RPSNC performs better than MST_1TRNP due to the reason explained above. However, QTA and FeSTA outperform RPSNC because they require more relay nodes than RPSNC.

#### 5.1.2. Pentagram Network

**Figure 12:**
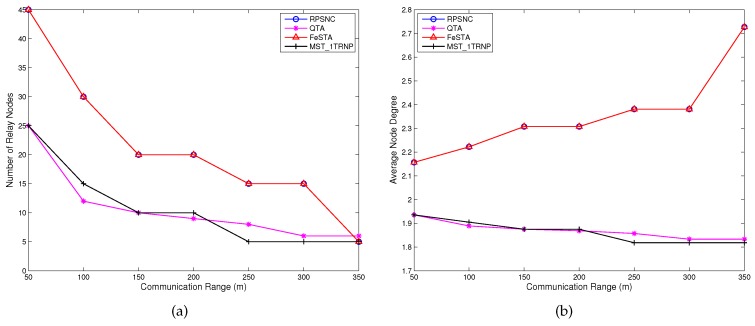
(**a**) Comparing the relay nodes count performance of RPSNC to the baseline approaches, with varying *R* in Pentagram network; (**b**) the average node degree of RPSNC and the baseline approaches, with varying *R* in Pentagram network.

**Number of relay nodes**: Figure 12a illustrates the performance comparison in terms of the number of relay nodes required under varying R. The figure shows that for all compared algorithms, when *R* increases, the number of required relay nodes reduces such that for R = 350 m, each of RPSNC, FeSTA and MST_1TRNP requires five relay nodes, in contrast to QTA that requires six relay nodes. This can be attributed to the fact that fewer relay nodes are required to connect the partitions when *R* increases. Hence, depending on the topology, both QTA and FeSTA may be superior to one another, since QTA outperformed FeSTA for the tested case of random networks. RPSNC and FeSTA achieve the same results, since the optimal relay nodes for the Pentagram network are placed at the Steiner points of the triangles (see Figure 1c).

**Average node degree**: The performance comparison of RPSNC, QTA, FeSTA and MST_1TRNP in terms of average node degree is illustrated in Figure 12b. Both RPSNC and FeSTA achieve the best results since they require more relay nodes than QTA and MST_1TRNP for R < 350 m. Interestingly, for R = 350 m, both RPSNC and FeSTA require fewer relay nodes than QTA and same relay nodes as MST_1TRNP and still outperform QTA and MST_1TRNP. This can be attributed to the fact that RPSNC and FeSTA place five relay nodes, each having a node degree of three to the Steiner points of the triangles (see Figure 1c), while QTA places three relay nodes, each having a node degree of three to the Steiner points of the quadrilaterals and three relay nodes, each having a node degree of two at the MST edges. As for MST_1TRNP, it places five relay nodes, each having a node degree of two along the MST edges.

## 6. Conclusions

In this paper, we have presented RPSNC, a novel polynomial time min-cost multicast relay nodes placement algorithm based on space network coding that aims to minimize the number of required relay nodes for connecting multiple disjoint WSN partitions. RPSNC generates the candidate relay nodes using non-uniform partitioning and Delaunay triangulation techniques, after which linear programming is applied for choosing the optimal relay nodes and computing their connection links with the terminals. Since the distance between the optimal relay nodes and terminals may exceed the communication range of the relay nodes, RPSNC populates extra coverage relay nodes along the connection links to achieve a strong connectivity. We investigated different performance metrics to evaluate the quality of the formed topologies. The simulation results have demonstrated that RPSNC significantly outperforms contemporary heuristics in the literature, not only in terms of the number of required relay nodes, but also in terms of the degree of connectivity of the formed topology and balanced traffic load. Our future work will focus on extending RPSNC to 3D WSNs. 

## Figures and Tables

**Table 1:** Comparison of some contemporary heuristic algorithms for connectivity restoration in WSNs through relay nodes placement.

Name	Objective (Other Than Connectivity)	Approach	Degree of Connectivity	Model	Time Complexity
QTA [6]	Minimizing relay node count	Centralized	1	Each partition is represented by a single node	$O(N^{4}logN)$
SMST * [7]					O(NlogN)
SMT-MSP ${}^{†}$ [9]					$O(N^{3})$
FeSTA [3]					$O(N^{4})$
SpiderWeb [11]	Providing high-quality topologies	Centralized/Distributed	1 and 2		$O\left(NlogN\left\lceil \frac{d}{R}\right\rceil \right)$
CIST ${}^{‡}$ [23]	Minimizing relay node count	Centralized	1	Each partition is represented by multiple nodes	Not available
CORP [4]	Providing high-quality topologies	Centralized/Distributed		Each partition is represented by a cell	O(r.N)

* Steinerized Minimum Spanning Tree; ${}^{†}$ Steiner Minimum Tree with Minimum number of Steiner Points; ${}^{‡}$ Connected Inter-Segment Topology.
